# Familial Budd-Chiari Syndrome in China: A Systematic Review of the Literature

**DOI:** 10.1155/2013/763508

**Published:** 2013-02-28

**Authors:** Xingshun Qi, Juan Wang, Weirong Ren, Ming Bai, Man Yang, Guohong Han, Daiming Fan

**Affiliations:** ^1^Xijing Hospital of Digestive Diseases, Fourth Military Medical University, Xi'an 710032, China; ^2^Department of Gastroenterology, No. 463 Hospital of Chinese PLA, Shenyang 710032, China

## Abstract

Familial occurrence of Budd-Chiari syndrome (BCS) has been reported in scattered cases, which potentially favors the congenital theory. A review of the literature was conducted to demonstrate this phenomenon in China. PubMed, VIP, and CNKI databases were searched for studies describing at least two Chinese BCS patients from the same one family. In the 18 eligible papers, 30 siblings or first-degree relatives from 14 families were diagnosed with BCS at 9 different centers. Common clinical presentations included varices of abdominal wall and lower limbs, edema of legs, and ascites. Type and location of obstruction were similar among these patients from the same one family. Screening for BCS was conducted in 65 family members from 3 families, demonstrating that 2 asymptomatic siblings from one family were further diagnosed with BCS. Factor V Leiden mutation was found in 3 of 4 patients from one family and in one of 2 patients from another one family. Prothrombin G20210A gene mutation was found in none of the 4 patients from the 2 families. In conclusion, our study showed the possibility of familial aggregation in Chinese BCS patients, but these available data cannot support the previous hypothesis that familial BCS originates from congenital vascular malformation.

## 1. Introduction

Thrombotic risk factors for Budd-Chiari syndrome (BCS) have been clearly recognized in Western countries [1, 2]. Major causal factors include myeloproliferative neoplasm (MPN), factor V Leiden (FVL) mutation, prothrombin G20210A gene mutation, antiphospholipid antibodies, hyperhomocysteinemia, paroxysmal nocturnal hemoglobinuria, antithrombin (AT), protein C (PC), protein S (PS) deficiencies, and others [1, 2]. Several European cohort studies have demonstrated that at least one underlying thrombotic risk factor is found in more than 80% of Western BCS patients [3, 4]. Accordingly, the recent American Association for the Study of Liver Diseases (AASLD) practice guideline has recommended that routine screening for these risk factors should be performed in BCS patients [2]. By contrast, a recent systematic review and meta-analysis of observational studies did not confirm the role of inherited AT, PC, or PS deficiency in the pathogenesis of BCS [5]. Additionally, the prevalence of other thrombotic risk factors for BCS appears to be very low in Chinese patients [6, 7]. For example, JAK2V617F mutation, a critical diagnostic marker of MPN, exists in approximately 37% of Western BCS patients [8], but in only 4% of Chinese BCS patients [9]. Other studies have reported that FVL mutation, prothrombin G20210A gene mutation, or paroxysmal nocturnal hemoglobinuria is extremely rare in Chinese patients with sporadic BCS [10–12]. These inconsistent results prompt us to explore the actual etiology of BCS in China.

Although the congenital theory for the pathogenesis of BCS is hypothesized by early histopathological studies [13–15], it has never been advocated by the current practice guideline and consensus due to the rarity of BCS in childhood and the anatomic and topographic inconsistency of BCS [16–18]. Several scattered case reports have demonstrated familial clustering of BCS, suggesting the possibility of congenital etiology [19–22]. In 1988, a French team reported 2 Italian sisters diagnosed with BCS [19]. Of note, the age at their first symptoms was 1.2 and 1.8 years old. In 1990, an Israeli study also demonstrated that one 30-year-old man and his older brother were simultaneously diagnosed with BCS, but his other first-degree relatives were asymptomatic without any imaging evidence of hepatic vein or inferior vena cava obstruction [20]. In 1995, a Dutch study further showed that 3 of 11 siblings from a single family were diagnosed with membranous obstruction of the inferior vena cava during early adult life [21]. In addition, a Japanese National survey revealed that one of 157 Japanese BCS patients had a family history of BCS [22]. Recently, a simultaneous occurrence of BCS was reported in two American twin sisters of 13 years of age with heterozygous FVL mutation [23]. 

In this paper, we have conducted a systematic review of the published literature to better understand the familial occurrence of BCS in Chinese patients.

## 2. Methods

### 2.1. Literature Search

The literature search has been described in detail elsewhere [24]. One database of English journals (i.e., PubMed database) and two databases of Chinese journals (i.e., Chinese Scientific and Technological Journal database (VIP database) and China National Knowledge Infrastructure database (CNKI database)) were employed for the review. VIP and CNKI databases were subscribed by our University library. For the PubMed database, search items were listed as follows: (“Budd Chiari” [All Fields]) AND (“China” [All Fields]). Two Chinese names of BCS (i.e., “Bu Jia Zong He Zheng” and “Bai Cha Zong He Zheng”) were also used as search items in the two Chinese databases. For the VIP database, search items were listed as follows: (“Budd Chiari” [All Fields]) OR (“Bu Jia Zong He Zheng” [All Fields]) OR (“Bai Cha Zong He Zheng” [All Fields]). Because there is no tag term of [All Fields] in the search strategy of CNKI database, search items for the database were listed as follows: (“Budd Chiari” [Keywords]) OR (“Budd Chiari” [Title]) OR (“Bu Jia Zong He Zheng” [Keywords]) OR (“Bu Jia Zong He Zheng” [Title]) OR (“Bai Cha Zong He Zheng” [Keywords]) OR (“Bai Cha Zong He Zheng” [Title]). The last search was performed on February 20, 2012.

### 2.2. Literature Selection

Two authors (X. Qi and W. Ren) independently reviewed abstracts and full texts of all identified papers to avoid omitting the information of family history. Inclusion criteria were as follows: (1) the BCS patients were Chinese and (2) at least two siblings or first-degree relatives in the same one family were simultaneously diagnosed with BCS. Exclusion criteria were as follows: (1) the patients were not diagnosed with BCS; (2) animal study; and (3) the patients were not from China. In cases of disagreement among the two reviewers, a consensus was achieved through discussion.

### 2.3. Data Extraction

A data extraction sheet was developed that included the journal, publication year, authors, affiliation, provinces, or regions where the data was collected, period of enrollment, demographic data (age and sex) of patients, standards of living, clinical presentations, types of BCS, results of family study, results of thrombotic risk factors for BCS, and outcomes of these patients. If the data was unavailable in some included papers, one reviewer (G. Han) would contact these principal investigators or their colleagues who participated in these studies by telephone. If these investigators agreed to provide accurate data, the requisite data for the present review were sent to them via a mobile phone short message.

### 2.4. Study Quality

Study quality was assessed by two reviewers (X. Qi and W. Ren). Discrepancies in the interpretation were resolved by consensus. The studies were considered of higher quality if they fulfilled the following predetermined criteria: (1) the demographic data (age and sex) were clearly described; (2) the diagnostic methods of BCS were clearly described; (3) the type and location of obstruction were recorded; and (4) screening for BCS in family members was done.

## 3. Results

### 3.1. Description of Included Studies

Overall, 112, 1332, and 1561 papers were identified in PubMed, VIP, and CNKI databases, respectively. Among them, 18 papers (i.e., one English-language paper and 17 Chinese-language papers) were eligible for the review. After duplicated publication was excluded, a total of 30 siblings or first-degree relatives from 14 Chinese families were diagnosed with BCS at 9 different centers (Table 1). These centers were in 6 provinces or regions (i.e., Beijing, Guangdong, Henan, Liaoning, Shaanxi, and Shandong provinces). Seven studies including only 3 families, in which demographic data, diagnostic methods of BCS, type and location of obstruction, and family screening were available, were considered of higher quality.

### 3.2. Clinical Presentation

The standards of living were available in only two patients from the 3rd family. Both of them were farmers with low incomes. Presenting symptoms were available in 14 BCS patients from 6 families. They included abdomen distension (*n* = 10), swelling of lower limbs (*n* = 8), variceal bleeding (*n* = 2), and no symptom (*n* = 2). Physical examination was available in 18 BCS patients from 8 families. They included varicosity of chest and abdominal wall and/or lower limbs (*n* = 9), hepatomegaly (*n* = 9), edema of legs (*n* = 6), ascites (*n* = 6), and splenomegaly (*n* = 3).

### 3.3. Type of BCS

Type and location of obstruction were reported in 22 BCS patients from 10 families. Inferior vena cava (IVC) and hepatic vein (HV) obstruction was found in 19 and 7 patients, respectively. Among them, both IVC and HV were obstructed in 4 patients. Generally, anatomical and topographic lesions were similar among these patients from the same one family.

### 3.4. Family Studies

**Figure 1:**
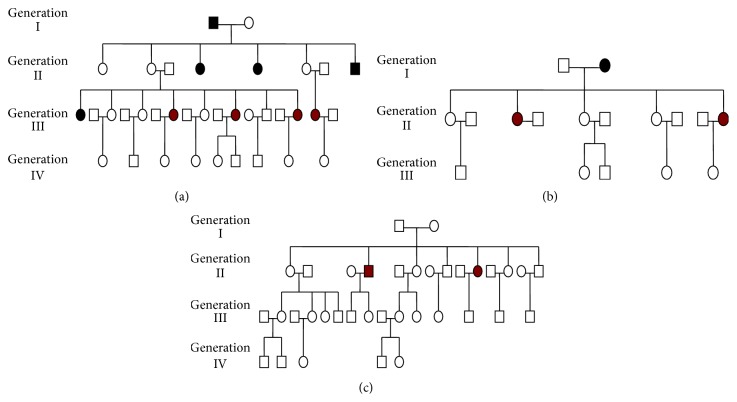
The genealogical charts of the 8th (a), 9th (b), and 10th (c) families. Circle and square represent female and male family members, respectively. Black bottom represents dead family member; red bottom represents BCS patients.

Familial screening for BCS was performed in 3 families (8th, 9th, and 10th families). In the 8th family, 27 additional family members from 4 generations were screened for BCS by color Doppler ultrasound. Of them, 14 and 13 were male and female, respectively. Age ranged from 8 to 94 years. Except for two symptomatic probands, two asymptomatic sisters were further diagnosed with BCS (Figure 1(a)). In the 9th family, 14 additional family members from 3 generations were screened for BCS by color Doppler ultrasound. Of them, 9 and 5 were male and female, respectively. Age ranged from 1 to 67 years. Except for two symptomatic probands, no other family member was diagnosed with BCS (Figure 1(b)). In the 10th family, 24 additional family members from 4 generations were screened for BCS by B-mode ultrasound. Of them, 10 and 14 were male and female, respectively. Age ranged from 3 to 78 years. Except for two symptomatic probands, no other family member was diagnosed with BCS (Figure 1(c)).

### 3.5. Thrombotic Risk Factors

Factor V Leiden (FVL) mutation was detected in 10 patients from the 7th, 8th, 12th, and 13th families. Three of 4 patients from the 7th family had heterozygous FVL mutation, one of two patients from the 8th family had heterozygous FVL mutation, and none of patients from the 12th and 13th families had FVL mutation. Prothrombin G20210A gene mutation was detected in 4 patients from the 12th and 13th families. But none of patients had this mutation. No other thrombotic risk factors were detected in these patients.

## 4. Discussion

To date, the etiological factors for most of Chinese BCS patients are still unclear because thrombotic risk factors for BCS are rarely found in China [6, 7, 9–12]. The present study shows familial occurrence of BCS in China, which potentially suggests that family history of BCS patients should be carefully inquired and the patient's first-degree relatives might be screened. But these available data cannot support the previous hypothesis that familial BCS is associated with the congenital vascular malformation. The main reasons for this consideration are as follows. First, although BCS patients from the same one family have the similar anatomical and topographic lesions, nearly all of these familial BCS cases are identified during adulthood rather than childhood. The fact indicates that these cases may be not of a congenital origin. However, we would like to emphasize that imaging evaluation for HV and IVC in these patients during the neonatal period is lacking and unrealistic. Therefore, we cannot exclude the possibility that BCS is unrecognized in childhood and the overt clinical presentations occur in adulthood. Indeed, if screening for BCS was not performed in the 8th family, two asymptomatic BCS patients would not be identified until their clinical presentations occurred. Second, an incomplete screening for inherited thrombotic risk factors in four families have demonstrated that FVL mutation is found in 4 of 6 familial cases from the 8th and 9th families. The phenomenon indicates that these cases may be caused by familial thrombophilia. Certainly, a complete work-up for thrombophilia should be adopted in further studies to evaluate the frequency of thrombotic risk factors in familial BCS patients. Third, the most possible causes for membranous obstruction of IVC (MOVC) in developing and underdeveloped countries so far are often considered an extremely poor standard of living and suspected bacterial infections in such poor living environment [18, 25]. Thus, it can be expected that familial aggregation in patients with MOVC may be associated with such factors. However, only one included study reported that two BCS patients from the same one family were farmers with low incomes. Therefore, whether or not a poor standard of living plays a role in familial occurrence of BCS deserves further investigation. This question is very important because health policy should focus on improving the standard of living in these patients' offspring, thereby preventing the development of BCS.

Strengths of the study are that literature searches were comprehensive, and all full-texts were reviewed to avoid omitting the information of familial BCS. Additionally, several limitations of this study need to be clarified. First, only a part of these included studies were of higher quality. The fact was primarily because the objectives of most studies were not to demonstrate familial occurrence of BCS, but to explore the outcome of BCS patients. And family screening was often difficult to be performed. Additionally, although we have tried our best to contact with the authors or their colleagues to collect more adequate information about these patients, only a few data could be acquired. Second, common causes of familial thrombophilia include FVL mutation, prothrombin G20210A gene mutation, and inherited AT, PC, and PS deficiencies [26]. If these familial BCS patients had a common inherited thrombophilia, the congenital malformation theory would not be approved. But screening for familial thrombophilia was incomplete in these included studies, and thrombotic risk factors were detected in only a minority of cases (i.e., FVL mutation was detected in 10 patients from 4 families, and prothrombin G20210A gene mutation was detected in 2 patients from 2 families). It might be explained by the fact that the role of thrombotic risk factors for BCS was not well established in China. Further, because FVL mutation and prothrombin G20210A gene mutation are rarely found in Chinese healthy population or patients with venous thromboembolism, AT, PC, and PS deficiencies are more common than the Western populations [27–30]; we recommend that screening for inherited AT, PC, and PS deficiencies appears to be more necessary in these patients.

In conclusion, our study confirmed the possibility of familial BCS in China, but it appeared that familial occurrence of BCS was not related to congenital vascular malformation. Familial thrombophilia and standard of living in these patients should be evaluated in further observational studies.

## Figures and Tables

**Table 1:** Familial Budd-Chiari syndrome in China—an overview of published literatures.

Authors	Journal (year)	Affiliation (province)	Number family	BCS patients	Age (years)	Clinical presentation	Type of BCS
Wang et al.	Curr Probl Surg (1996) EN	Beijing Post and Telecommunication Hospital (Beijing)	1st family	Two brothers	NA	NA	IVC webs
			2nd family	Twin sisters	NA	NA	MOVC

Sun et al.	Gan Dan Wai Ke Za Zhi (1997) CN	No. 89 Hospital of Chinese PLA (Shandong)	3rd family	Two brothers (farmers)	24	Abdominal distension, swelling of lower limbs (4 yrs), ascites, hepatomegaly, varicosity of abdominal wall and lower limbs	IVC stenosis with MHV obstruction
					26	Abdominal distension and swelling of lower limbs (6 yrs), ascites, hepatomegaly, splenomegaly, varicosity of abdominal wall and lower limbs	IVC stenosis with MHV obstruction

Liu et al.; Wang et al.	Zhonghua Xiao Hua Za Zhi (1999); The 7th Chinese National Congress of Gastroenterology (2007); Wei Chang Bing Xue (2007) CN	Departments of Digestive Diseases and Interventional Radiology, General Hospital of Chinese PLA (Beijing)	4th family	An elder brother and a younger sister	29	Abdominal distension (20 days), oliguria and swelling of lower limbs (4 days), gross ascites, hepatomegaly, varicosity of chest and abdominal wall	Three HVs obstruction
					25	Abdominal distension (2 months); hepatomegaly, ascites	Three HVs obstruction with IVC stenosis
			5th family	Two brothers	NA	Hepatomegaly	HV obstruction

Liu et al.	Lin Chuang Xiao Hua Bing Za Zhi (2000) CN	Second People's Hospital of Anyang City (Henan)	6th family	Father and an elder son	NA	NA	NA
			7th family	An elder brother and a younger sister	NA	NA	NA

Feng et al.	Zhonghua Yi Xue Za Zhi (2000); Zhonghua Xue Ye Xue Za Zhi (2000); Zhonghua Fang She Za Zhi (2001) CN	Department of Radiology, First Affiliated Hospital of China Medical University (Liaoning)	8th family	Four sisters	40∗	Brachypnea (15 yrs), abdominal distension and swelling of lower limbs (10 yrs), varicosity of abdominal wall and lower limbs	SOVC
					28∗	Swelling of lower limbs (5 yrs), varicosity of chest and abdominal wall and lower limbs, hepatomegaly	MOVC
					35∗	Asymptomatic	IVC stenosis
					23	Asymptomatic	IVC stenosis with two HVs stenosis
			9th family	Two sisters	43	Swelling of lower limbs (4 yrs), ascites, varicosity of abdominal wall and lower limbs	SOVC with IVC thrombosis
					33∗	Abdominal distension and swelling of lower limbs (5 yrs), varicosity of chest and abdominal wall, hepatomegaly	MOVC

Wang et al.	Guang Dong Yi Xue Za Zhi (2000); Shi Yong Yi Xue Za Zhi (2001); Zhongguo Xian Dai Yi Xue Za Zhi (2001); Lin Chuang Gan Dan Bing Za Zhi (2005) CN	Department of Digestive Diseases, Second Affiliated Hospital of Ji'nan University (Guangdong)	10th family	An elder brother and a younger sister	50	Recurrent variceal bleeding (30 yrs), abdominal distension, hepatomegaly, no ascites	SOVC
					43	Abdominal distension (1 yr); normal liver and spleen, no ascites	MOVC

Yuan et al.; Qin et al.	Hua Bei Mei Tan Yi Xue Yuan Xue Bao (2002); Zhongguo Pu Tong Wai Ke Za Zhi (2002) CN	Department of General Surgery, First Affiliated Hospital of Xinxiang Medical College (Henan)	11th family	An elder sister and a younger sister	NA	Asymptomatic, esophageal varices	MOVC

Zhao et al.	Zhonghua Xiao Hua Za Zhi (2003) CN	Hospital of Chenggu County (Shaanxi)	12th family	An elder sister and a younger brother	36	Abdominal distension and swelling of lower limbs (8 yrs), varicosity of chest and abdominal wall, splenomegaly, normal liver, no ascites	IVC obstruction
					31	Abdominal distension (0.5 yr), massive variceal bleeding (4 days), edema of both lower limbs, varicosity of chest and abdominal wall and lower limbs, splenomegaly, normal liver, moderate ascites	IVC stenosis

Zheng et al.; Lin et al.	Zhengzhou Da Xue Xue Bao (2005); Zhonghua Pu Tong Wai Ke Za Zhi (2006) CN	Department of General Surgery, First Affiliated Hospital, Zhengzhou University (Henan)	13th family	An elder sister and a younger brother	NA	NA	NA
			14th family	Twin sisters	NA	NA	NA

Notes: ^*^represents that the patient had factor V Leiden mutation.

AD: abdominal distension; BCS: Budd-Chiari syndrome; CN: Chinese journal; EN: English journal; F: female; HV: hepatic vein; IVC: inferior vena cava; M: male; MHV: middle hepatic vein; MOVC: membranous obstruction of inferior vena cava; NA: not available; SLL: swelling of lower limbs; SOVC: segmental obstruction of inferior vena cava; VAWLL: varicosity of abdominal wall and lower limbs; VB: variceal bleeding.

## Abbreviations

AT: Antithrombin
BCS: Budd-Chiari syndrome
CNKI: China National Knowledge Infrastructure
FVL: Factor V Leiden
HV: Hepatic vein
IVC: Inferior vena cava
MOVC: Membranous obstruction of inferior vena cava
MPN: Myeloproliferative neoplasm
PC: Protein C
PS: Protein S.
